# A Rational Approach to Estimating the Surgical Demand Elasticity Needed to Guide Manpower Reallocation during Contagious Outbreaks

**DOI:** 10.1371/journal.pone.0122625

**Published:** 2015-04-02

**Authors:** Hsiao-Mei Tsao, Ying-Chou Sun, Der-Ming Liou

**Affiliations:** 1 Institute of Biomedical Informatics, National Yang-Ming University, Taipei, Taiwan; 2 School of Medicine, National Yang-Ming University, Taipei, Taiwan; 3 Department of Radiology, Taipei Veterans General Hospital, Taipei, Taiwan; University of Massachusetts, UNITED STATES

## Abstract

**Background:**

Emerging infectious diseases continue to pose serious threats to global public health. So far, however, few published study has addressed the need for manpower reallocation needed in hospitals when such a serious contagious outbreak occurs.

**Aim:**

To quantify the demand elasticity of the major surgery types in order to guide future manpower reallocation during contagious outbreaks.

**Materials and Methods:**

Based on a nationwide research database in Taiwan, we extracted the monthly volumes of major surgery types for the period 1998–2003, which covered the SARS period, in order to carry out a time series analysis. The demand elasticity of each surgery type was then estimated by autoregressive integrated moving average (ARIMA) analysis.

**Results:**

During the study period, the surgical volumes of most selected surgery types either increased or remained steady. We categorized these surgery types into low-, moderate- and high-elastic groups according to their demand elasticity. Appendectomy, ‘open reduction of fracture with internal fixation’ and ‘free skin graft’ were in the low demand elasticity group. Transurethral prostatectomy and extracorporeal shockwave lithotripsy (ESWL) were in the high demand elasticity group. The manpower of the departments carrying out the surgeries with low demand elasticity should be maintained during outbreaks. In contrast, departments in charge of surgeries mainly with high demand elasticity, like urology departments, may be in a position to have part of their staff reallocated.

**Conclusions:**

Taking advantage of the demand variation during the SARS period in 2003, we adopted the concept of demand elasticity and used a time series approach to figure out an effective index of demand elasticity for various types of surgery that could be used as a rational reference to carry out manpower reallocation during contagious outbreak situations.

## Introduction

In the past few decades, emerging infectious diseases, such as SARS (Severe Acute Respiratory Syndrome), have posed serious threats to global public health [1]. With rapid spread and high mortality rates, these diseases often lead to tremendous public fear [2, 3]. Ample studies have been reported concerning effective disease control in hospitals in such circumstances [4–7]. Nevertheless, to our best knowledge, there is few published study on how to reallocate manpower in hospitals when facing the disastrous contagious outbreaks [8]. While healthcare providers are at the high risk of infection, unnecessary staffing is inevitably to raise the risk of disease transmission. It is therefore mandatory for healthcare institutions to reallocate manpower from less in demand services to critical ones.

In this study, we adopted the concept of demand elasticity from economics in order to analyze demand elasticity for major surgery categories. In economics, “elasticity” refers to the degree of responsiveness to a specific factor and “demand elasticity” refers to the changes in demand quantity in response to a specific factor, such as price, income, or risk. Nevertheless, it is essentially difficult to estimate the demand elasticity of medical services since both supply and demand are usually quite stable in the healthcare market. One of the common exceptions to bring about fluctuations is a natural catastrophe (such as Hurricane Katrina in New Orleans). Once it occurs, both supply and demand may change. During the SARS epidemic in Taiwan, in contrast, however, the supply of medical services remained steady, while the demands for medical services decreased dramatically due to public fear [2, 9, 10]. Hence, medical claim data across the SARS period should be able to serve as the correct basis to estimate the demand elasticity of various types of medical services.

We took advantage of the medical claim data across the period of SARS outbreak in Taiwan and applied time series analysis in order to estimate the demand elasticity of surgery. Time series analysis is used in various scientific fields to uncover hidden patterns or to forecast future values; it requires a sequence of data points that are collected at consecutive uniform time intervals. Thus, we used monthly surgical volumes as the time series data and calculated mean absolute percentage error (MAPE) to serve as an index of demand elasticity.

In this paper, we created an indicator for demand elasticity that addresses three goals: (1) to identify the demand elasticity of selected major surgery categories; (2) to categorize different surgeries according to their demand elasticity; and (3) to create guidance for manpower reallocation during contagious outbreaks. According to the results of time series analysis, we categorized the resulting index values, based on demand elasticity, into three groups, namely low, moderate and high demand elasticity. Surgeries with low demand elasticity refer to those always in a consistent service demand. In contrast, the service demand of surgeries with high demand elasticity fluctuates dramatically. Next, we used demand elasticity to categorize the various types of surgery. The demand elasticity indices for each surgical category can be applied to properly reallocate staffing during contagious disease outbreaks in the future.

## Materials and Methods

The National Health Insurance (NHI) program in Taiwan was launched in 1995. It covered 93% of population in Taiwan in 1997 and kept increasing thereafter to 99% by the end of 2010. The National Health Insurance Research Database (NHIRD) is a nationwide database extracted from the claim data of the NHI program for research purposes [11]. This anonymous database contains information regarding inpatient and outpatient medical claims, including prescription records. In this study, we used a longitudinal cohort dataset (Longitudinal Health Insurance Database, LHID2005) that contains claim data of one million randomly sampled individuals who were insured in 2005 [12]. This dataset has been confirmed as having no significant differences in either age, gender or health care costs from its whole population composed of all beneficiaries under the NHI program.

In this study, we used inpatient claim data retrieved between 1998 and 2003 from LHID2005. Inpatient records comprise administrative information including admission month, year, admission type, and various demographic information such as age, gender, and medical costs as well as medical information such as diagnosis and procedure carried out. ICD9-CM procedure codes are used to code procedures and surgery in this database. The first two digits represent general procedures, followed by two digits that describe in more detail the specific subtype of the general procedure. For example, ICD9-CM procedure codes 47.0, 47.01 and 47.02 represent appendectomies, laparoscopic appendectomies and other appendectomies, respectively. Thus the last two codes provide detailed information about the specific surgical technique employed. Based on our research purpose, we used the first three digits of the ICD9-CM procedure codes only; thus in the example above, 47.01 and 47.02 were represented in our study by 47.0X.

We conducted ARIMA analysis to estimate the demand elasticity of various selected major surgery categories performed by obstetricians, urologists, orthopedists, gastroenterologists, colorectal surgeons, neurosurgeons and general surgeons as listed in Table 1. For each surgery type, we first used the monthly surgical volume from 1998 to 2001 as time series data to identify the best ARIMA model, namely the one with the lowest AIC (Akaike information criterion). Then, a Q-test was performed to see if the error was autocorrelated. Next the dataset for 2002 was used as an out-of-sample dataset for model selection and evaluation, and MAPE was used to estimate the prediction power. MAPE is the average difference between observed volume and predicted volume, divided by observed volume. For all out-of-sample evaluations, the MAPE values for all models were less than 25%. The augmented Dickey–Fuller (ADF) test is a unit root test performed to examine whether the data were stationary. Lastly, after model evaluation, the time series data from 1998 to 2002 were used to forecast surgical volume in 2003. The observed data and expected data were plotted along with their 95% confidence intervals. The SARS epidemic in Taiwan started in March 2003 and lasted until July 2003. During this period, the needs for medical services decreased dramatically, particularly in May [10]. Therefore, we defined the MAPE value for May 2003 as an index of demand elasticity. All *p* values of <0.05 were considered to be statistically significant.

**Table 1:** Monthly volumes and demand elasticity of selected surgery in LHID2005.

ICD9-CM procedure code		Monthly volume	Demand elasticity
Codes	Description	(mean ± SD)	
79.3X	Open reduction of fracture with internal fixation	257.1±32.2	26.4
49.4X	Procedures on hemorrhoids	85.8±18.0	108.2
47.0X	Appendectomy	85.5±11.4	10.0
51.2X	Cholecystectomy	56.5±12.8	112.3
68.4X	Total abdominal hysterectomy	48.5±13.6	76.2
86.6X	Free skin graft	47.3±8.8	27.6
80.5X	Excision, destruction and other repair of intervertebral disc	40.6±11.0	76.3
54.5X	Lysis of peritoneal adhesions	40.2±9.2	73.4
60.2X	Transurethral prostatectomy	39.4±9.6	279.6
68.5X	Vaginal hysterectomy	37.1±10.3	120.1
98.5X	Extracorporeal shockwave lithotripsy	32.2±8.3	196.5

All claim data in the research database, which we applied for this study, were anonymized and de-identified before its release. According to the local regulations, informed consent is not required in this situation. This study was reviewed and exempted from full review by the institutional review board of Taipei Veterans General Hospital, Taipei, Taiwan.

## Results

**Fig 1:**
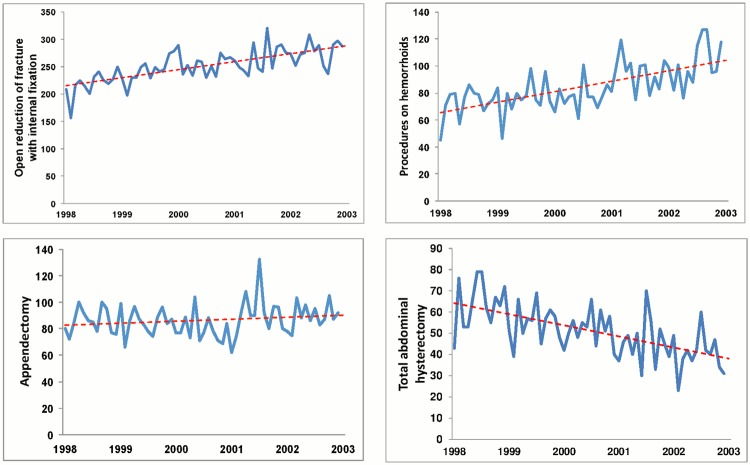
Trends in surgical volumes over the period 1998–2002. (A) Open reductions of fracture (B) Procedures related to hemorrhoids (C) appendectomies, and (D) total abdominal hysterectomies.

During the study period, the surgical demands grew steadily with the expanding and aging population of Taiwan. The surgical volumes of the selected surgery types either increased or remained steady over time, with the exception of total abdominal hysterectomy (ICD9 code 68.4X) (Fig 1). Several studies have reported that vaginal hysterectomy is safer and has fewer complications than abdominal hysterectomy, thus, the route for carrying out hysterectomy is shifting toward the vaginal approach. In fact, the total volume for hysterectomies showed a growing trend over the study period.

**Fig 2:**
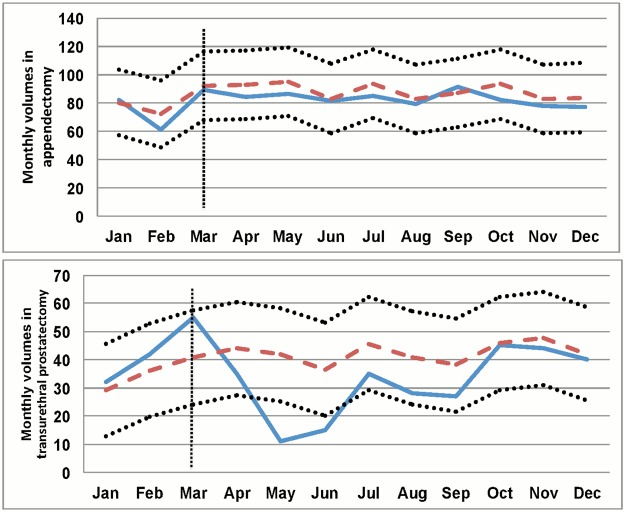
Observed and predicted monthly volumes for various surgeries in 2003. Monthly volume (y axis) is plotted against time (x axis). The solid line shows the observed values. The dashed line shows the predicted values and 95% confidence intervals (dotted lines). The vertical dotted line indicates the period with an outbreak of SARS in Taiwan. (A) During the SARS epidemic, the observed surgical volume for appendectomy remained stable. (B) The observed volume for transurethral prostatectomy during the SARS epidemic decreased dramatically, which implies that transurethral prostatectomy has a high demand elasticity.

Table 1 shows the monthly volume and the demand elasticity of each selected surgery type. We arbitrarily classified the surgery types into three categories according to their demand elasticity indices. Indices below 30, in the range 30–190 and above 190, represented low, moderate and high demand elasticity, respectively. Appendectomy (ICD9 code 47.0X) was found to have the lowest elasticity index, followed in order by ‘open reduction of fracture with internal fixation’ (ICD9 code 79.3X) and ‘free skin graft’ (ICD9 code 86.6X) as having the next lowest elasticity indices. A low demand elasticity implies that the fear for SARS did not make any significant change in the elasticity and that the observed values for the relevant month, May 2003, fall into the expected range obtained using the ARIMA models. Fig 2A illustrates the monthly volume of appendectomies in 2003.

The high demand elasticity group consisted of transurethral prostatectomy (ICD9 code 60.2X) and extracorporeal shockwave lithotripsy (ICD9 code 98.5X). A high demand elasticity means that the needs for surgery were seriously impacted during the SARS period. Specifically, the observed monthly volumes, especially in April and May 2003, dropped much lowers than the expected values. Fig 2B illustrates the monthly volume of transurethral prostatectomies in 2003. The remaining surgery types all fell into the moderate demand elasticity group.

In a situation where an emerging infectious disease outbreak comes into an epidemic, hospitals need to reallocate some of their staff from their normal tasks to disease control and patient care. Since ESWL and transurethral prostatectomy have the high demand elasticity, relevant staff may be the suitable target to be reallocated., The manpower serving the surgeries with low demand elasticity should be maintained at least in a normal status. On the other hand, for surgical departments that fall into the moderate demand elasticity group, it will be necessary for institution managers to take additional factors into account before changing their manpower levels.

## Discussion

When facing an emerging infectious disease, we need to prepare ahead before the epidemic really out breaks, e.g. manpower reallocation planning and ward space preparation. The effect of traumatic events like a SARS epidemic is distinct from a natural catastrophe. Although the total casualties related to SARS were limited, the outbreak did cause tremendous panic in the public. In this study, we combined the concept of demand elasticity and the technique of time series analysis, and as a result were able to elucidate the demand elasticity for various specific surgeries. This provides us with information necessary to predict service demand during an infectious outbreak situation so that the real surgical demand elasticity based on quantitative evidence can be calculated. The strength of this study is that it was able to quantify surgical demand elasticity as a reference point for future manpower reallocation guidance.

From the general view of medical practice, our results are fairly rational. In essence, surgery with low demand elasticity is almost emergent in nature, as confirmed by our study that appendectomies, open reductions of fracture and free skin grafts are all the types of surgery present in low demand elasticity group. They are also emergencies needed to be done as soon as possible. In contrast, the types of surgery with high demand elasticity are usually elective in nature—the patients may go through quite a long period of time from occurrence of indication, being informed of the need for surgery, and the final action of surgery. Transurethral prostatectomy and ESWL, which are members of the high demand elasticity group in our study, are usually elective. The most common indication for transurethral prostatectomy is benign prostatic hyperplasia (BPH). Patients with BPH may have trouble with urination; for example, an urgency to urinate. Generally, the symptoms are tolerable without emergency unless there is urinary obstruction and infection. ESWL for urinary calculus is in similar case. Compared with the risk of contracting SARS, discomfort caused by BPH or urinary calculus is much less threatening. The patients would rather avoid receiving surgery during the SARS epidemic and thus decreased the demand for transurethral prostatectomy and ESWL.

A great number of reports have emphasized the need to increase the manpower supply and expand health services during contagious outbreaks, but these studies provide little recommendation or guidance on how such manpower reallocation can be achieved [4–7]. It is generally agreed that superfluous staffing should shift from the less demanding departments to the medical areas where extra manpower is urgently needed. In addition to the issue of surplus and shortage of manpower, the cost of resources repurposing should also be taken into account. As in our case, urologists are specialized for localized infection like UTI; they may not be competent enough to aid disease control. In order to help control the outbreak situation, they may need some specific training. This repurposing not only costs money but also takes time. Murphy *et*. *al*. have proposed a competency-based approach to health human resources planning. In their approach, the healthcare providers’ competence is the key component for manpower reallocation regardless of the service demand of the original settings. We suggest that surgery demand elasticity can serve as another dimension to optimize the practice of manpower reallocation during an infectious outbreak.

## Limitations

There are some limitations to this study. Firstly, this is just an observational study. Inevitably, extraneous factors may have affected the findings. In addition, the existing data may be incomplete, inaccurate, or inconsistently collected over time. Secondly, the volumes of surgery were analyzed using claim data. Therefore, if the surgery was not coded as major surgery during inpatient episodes, slight deviations from the real volumes of surgery might exist. Finally, the results from Taiwan's medical claim data may not be directly applicable to the situation in other countries.

## Conclusions

Taking advantage of the demand variation during the SARS epidemic period in 2003 in Taiwan, we used the concept of demand elasticity and the method of time series, to calculate elasticity indices for various types of surgery. The results are plausible from a medical point of view and therefore we believe that the demand elasticity index can be used as a rational reference for manpower reallocation during infectious outbreak situations.
